# Outcome after intracranial haemorrhage from dural arteriovenous fistulae; a systematic review and case-series

**DOI:** 10.1007/s00415-015-7898-x

**Published:** 2015-09-26

**Authors:** W. M. T. Jolink, J. M. C. van Dijk, C. J. J. van Asch, G. A. P. de Kort, A. Algra, R. J. M. Groen, G. J. E. Rinkel, C. J. M. Klijn

**Affiliations:** Department of Neurology and Neurosurgery, Brain Center Rudolf Magnus, University Medical Center Utrecht, Utrecht, The Netherlands; Department of Radiology, University Medical Center Utrecht, Utrecht, The Netherlands; Department of Neurosurgery, University Medical Center Groningen, University of Groningen, Groningen, The Netherlands; Department of Neurology, Donders Institute for Brain Cognition & Behaviour, Center for Neuroscience, Radboud University Medical Center, Nijmegen, The Netherlands; Julius Center for Health Sciences and Primary Care, University Medical Center Utrecht, Utrecht, The Netherlands

**Keywords:** Dural arteriovenous fistula, Intracranial haemorrhage, Outcome

## Abstract

Dural arteriovenous fistulae (DAVFs) are a rare cause of intracranial haemorrhage. We aimed to investigate outcome of patients with intracranial haemorrhage from a DAVF. We performed a systematic literature search for studies reporting outcome after intracranial haemorrhage caused by a DAVF. We used predefined selection criteria and assessed the quality of the studies. In addition, we studied outcome in all patients with DAVF who had presented with intracranial haemorrhage at two university centers in the Netherlands, between January 2007 and April 2012. We calculated case fatality and proportions of patients with poor outcome (defined as modified Rankin Scale ≥ 3 or Glasgow Outcome Scale ≤ 3) during follow-up. We investigated mean age, sex, mid-year of study and percentage of patients with parenchymal haemorrhage as determinants of case fatality and poor outcome. The literature search yielded 16 studies, all but two retrospective and all hospital-based. Combined with our cohort of 29 patients the total number of patients with DAVF-related intracranial haemorrhage was 326 (58 % intracerebral haemorrhage). At a median follow-up of 12 months case fatality was 4.7 % (95 % CI 2.5–7.5; 17 cohorts) and the proportion of patients with poor outcome 8.3 % (95 % CI 3.1–15.7; nine cohorts). We found no effect of mean age, sex, mid-year of the cohorts and percentage of patients with parenchymal haemorrhage on either outcome. Hospital based case-series suggest a relatively low risk of death and poor outcome in patients with intracranial haemorrhage due to rupture of a DAVF. These risks may be underestimated because of bias.

**Electronic supplementary material:**

The online version of this article (doi:10.1007/s00415-015-7898-x) contains supplementary material, which is available to authorized users.

## Introduction

Dural arteriovenous fistulae (DAVFs) are rare vascular lesions with a detection rate of 0.16/100,000 adults per year [1]. They represent 10 to 15 % of all intracranial vascular malformations [2]. About 20 % of patients with a DAVF present with intracranial haemorrhage [1, 3], which is in most patients associated with venous outflow from the DAVF into a cortical vein (cortical venous reflux) [4–7].

Information on outcome after intracranial haemorrhage due to a DAVF is limited due to the rarity of the lesions. In patients with intracerebral haemorrhage (ICH) due to rupture of an arteriovenous malformation (AVM), the risk of poor outcome (modified Rankin Scale (mRS) ≥ 3) is lower than after spontaneous ICH, even after taking into account predictors of outcome including age [8]. It is unclear whether outcome after ICH due to DAVF is similar to outcome after a rupture of an AVM or comparable to outcome after spontaneous ICH.

We aimed to investigate outcome after DAVF-related intracranial haemorrhage by a systematic review of the literature and studying a case-series in two tertiary referral centers in the Netherlands.

## Patients and methods

### Systematic literature search

We performed a systematic literature search using PubMed and Embase for studies of patients with a DAVF-related intracranial haemorrhage from January 1980 to April 2015 (Supplementary Fig. 1) according to the PRISMA statement methodology [9]. We used different combinations of ‘h(a)emorrhage’ or ‘hematoma’ and ‘dural arteriovenous fistula’ and its synonyms (see Supplementary Table 1 for details of the search strategy). References of included articles, related citations and relevant reviews were screened for additional articles. After filtering duplicates, articles were screened by one reviewer (WMTJ) on title and abstract using predefined inclusion and exclusion criteria. Inclusion criteria were: (1) sample size of at least ten patients with DAVF-related intracranial haemorrhage; (2) the diagnosis of DAVF had to be proven by digital subtraction angiography in at least 90 % of patients; (3) follow-up of at least one month; (4) if a study reported not only patients with DAVF-related intracranial haemorrhage but also other presenting symptoms, it had to be possible to extract data on outcome for the patients with DAVF-related intracranial haemorrhage separately. We excluded study cohorts when more than 10 % of the fistulae were located at the cavernous sinus, because these lesions have a different etiology, more commonly a traumatic cause, a mostly benign presentation, without intracranial haemorrhage, and a low rate of cortical venous reflux [10, 11]. We defined a study as high quality based on predefined criteria, namely, prospective, population-based, patients with intracranial haemorrhage as inception cohort, and clearly defined outcome measures (mRS or Glasgow Outcome Scale (GOS)). Two investigators (WMTJ and CJJvA) independently extracted data on study characteristics as summarized in Table 1 and Supplementary Table 2. In case of multiple publications from one cohort, we included the most suitable publication. We defined poor outcome as a mRS ≥ 3 or GOS ≤ 3. If a study used other outcome measures, we extracted data on patients with poor outcome or death according to the definition of the authors. Discrepancies in extracted data between the two investigators were dissolved by a third author (CJMK).

**Fig. 1:**
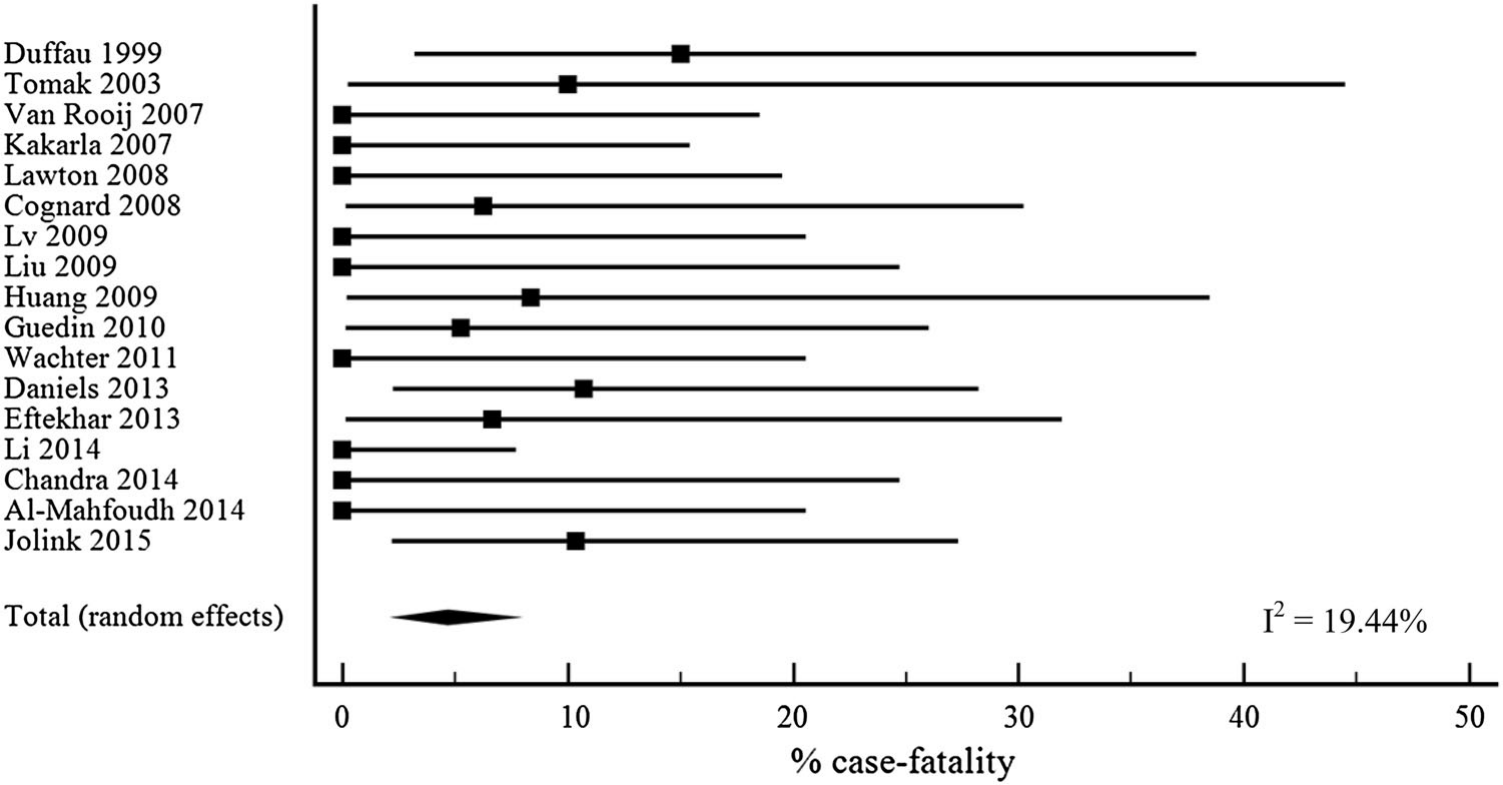
Case fatality with 95 % CI after DAVF-related intracranial haemorrhage

**Table 1:** Characteristics of cohorts of patients with DAVF-related intracranial haemorrhage

References	Design	Patients with DAVF-related intracranial haemorrhage (*n*)	Duration of FU (months, range)	Case fatality (%, 95 % CI)	Poor outcome (%, 95 % CI)
Duffau et al. [15]	RS	20	10 (0.3–25)	15 (3.2–38)	NR
Tomak et al. [16]	RS	10	11.5 (1–123)	10 (0.3–45)	10 (0.3–45)
Van Rooij et al. [17]	RS	18	1.5–3	0 (0–19)	NR
Kakarla et al. [18]	RS	22	12 (0–84)	0 (0–15)	0 (0–15)
Lawton et al. [19]	RS	17	50 (1–108)	0 (0–20)	0 (0–20)
Cognard et al. [20]	PS	16	3	6.3 (0.2–30)	NR
Lv et al. [21]	RS	16	8 (1–16)	0 (0–21)	0 (0–21)
Liu et al. [22]	RS	13	45 (2–84)	0 (0–25)	0 (0–25)
Huang et al. [23]	RS	12	3–24	8.3 (0.2–39)	8.3 (0.2–39)
Guedin et al. [24]	RS	19	3–6	5.3 (0.1–26)	NR
Wachter et al. [25]	RS	16	50 (2–120)	0 (0–21)	NR
Daniels et al. [26]	RS	28	17	10.7 (2.3–28)	28.6 (13–49)
Eftekhar and Morgan [27]	RS	15	18 (2–82)	6.7 (0.2–32)	NR
Li et al. [28]	RS	46	20	0 (0–8)	13 (5–26)
Chandra et al. [29]	RS	13	28 (12–63)	0 (0–25)	NR
Al-Mahfoudh et al. [30]	PS	16	67 (24–102)	0 (0–21)	NR
Jolink (2015)	RS	29	5 (0–12)	10 (2.2–27)	14 (3.9–32)

*CI* confidence interval, *DAVF* dural arteriovenous fistula, *FU* follow-up, *NR* not reported specifically for patients with a DAVF presenting with intracranial haemorrhage, *PS* prospective study design, *RS* retrospective study design

### Cohort study

From the prospectively kept Utrecht Stroke Database, including all patients with ischaemic stroke and intracerebral haemorrhage, and the Subarachnoid Haemorrhage Database, we retrieved all patients with first DAVF-related intracerebral (ICH), intraventricular (IVH) or subarachnoid haemorrhage (SAH), who presented to the University Medical Center Utrecht, the Netherlands, between January 2007 and April 2012. At the University Medical Center Groningen we retrieved data on patients from a prospectively kept neurovascular database, which we cross-checked with patient discharge records. We retrieved data on all patients with first DAVF-related intracranial haemorrhage who presented between January 2007 and January 2012.

For all patients baseline characteristics were collected as listed in Supplementary Table 2. We reviewed imaging studies for location and extension of haemorrhage (intracerebral, subarachnoid, subdural or intraventricular, or a combination of these) and location of the DAVF and we classified DAVF’s according to Borden-Shucart (GAPdK and JMCvD) [4]. The diagnosis of DAVF had to be proven by digital subtraction angiography. For each patient with an ICH we calculated the ICH score consisting of known predictors of poor outcome after ICH [12]. The ICH score is the sum of individual points assigned as follows: Glasgow Coma Scale (GCS) (3–4 = 2 points, 5–12 = 1 point, 13–15 = 0 points), age (≥80 years = 1 point, <80 years = 0 points), infratentorial origin (yes = 1 point, no = 0 points), ICH volume (≥30 cm^3^ = 1 point, <30 cm^3^ = 0 points) and presence of IVH (yes = 1 point, no = 0 points). We calculated ICH volume (mL) on brain imaging at presentation with the ABC/2 formula [13, 14]. In addition we retrieved information on death and poor functional outcome after at least one month. We defined poor outcome as a mRS ≥ 3.

### Statistical analysis

We calculated mean age, proportion of males, proportion of patients with an intracerebral component of the haemorrhage, average intracerebral haemorrhage volume (for patients with ICH), follow-up period, and proportion of patients who died or had a poor outcome with corresponding 95 % confidence intervals (CIs) during follow-up of at least one month. In the cohort study we performed univariate analysis of age, male sex, ICH volume > 30 mL, and presence of a parenchymal component of the haemorrhage for case fatality and poor outcome with Cox proportional hazards analysis, resulting in hazard ratios (HR) with 95 % CIs.

Proportions of patients with case fatality or poor outcome were pooled using MedCalc for Windows, version 12.7 (MedCalc Software, Ostend, Belgium). Heterogeneity between studies was calculated with the I^2^ statistic. To investigate the association of mid-year of study, mean age, percentage of males and percentage of patients with an parenchymal component of the haemorrhage in the study cohorts with the proportion of patients with case fatality or poor outcome, we performed linear regression analysis weighted by the inverse standard error.

## Results

The literature search yielded sixteen studies that described a total of 576 patients of whom 297 patients had a DAVF-related intracranial haemorrhage (Table 1) [15–30]. Two studies had a prospective design [20, 30]; all studies were hospital-based, and none fulfilled all the criteria for high-quality study. In all studies DAVFs were proven with angiography in 100 % of patients.

Three studies described patients with haemorrhagic presentation only [15, 26, 28], whereas all other studies were not restricted to patients with ruptured DAVF, but rather descriptions of cohorts of patients with DAVF irrespective of their presenting symptoms or treatment. One study described symptomatic DAVFs [16], ten studies described DAVFs treated by a specific modality (surgery, six studies [18, 19, 22, 25, 27, 30], endovascular treatment, four studies [20, 21, 23, 29]), and two studies specifically described patients with DAVFs with cortical venous reflux [17, 24].

Four studies used the mRS [19, 21, 26, 28], three studies the GOS [16, 18, 22] and one study described clinical outcome in terms of excellent, good and death [23]. Eight studies did not report on functional outcome. Information concerning cortical venous reflux, ICH volume and ICH score was not reported in most of the studies. Duration of follow-up varied between studies (Table 1). In the case-series, all patients with DAVF-related intracranial haemorrhage were included after their first haemorrhage; in two studies, a total of eight patients had a rebleed before treatment and outcome assessment [23, 28].

In the cohort study we included 29 patients; three patients died during follow-up (Supplementary Table 2). One patient (3 %) had a Borden–Shucart type 1 lesion, nine patients (31 %) a type 2 lesion and 19 patients (66 %) a type 3 DAVF. Eighteen patients presented with intracerebral haemorrhage. ICH volumes ranged between 0.2 and 46 ml. Five patients had an ICH score of 0 (28 %), six of 1 (33 %), three of 2 (17 %), three patients had an ICH score of 3 (17 %) and one patient of 4 (6 %). During follow-up none of the patients with an ICH score of 0, 1 or 2 died. One patient with an ICH score of 3 and one patient with an ICH score of 4 died. Two patients with an intracerebral haemorrhage had a poor functional outcome (both patients died). Eleven patients presented with a SAH or IVH or a combination; one patient with a SAH died during follow-up. Two patients with a SAH or IVH had a poor functional outcome (one died and the other had a mRS of 3).

We found no associations with case fatality for age (HR 1.2, 95 % CI 0.96–1.4), male sex (HR 0.4, 95 % CI 0.03–3.9), ICH volume > 30 mL (HR 6.1, 95 % CI 0.4–99.3), and a parenchymal component of the haemorrhage (HR 1.3, 95 % CI 0.1–14.3). We also found no associations for poor outcome (data not shown).

Combining the data from the literature search and our series, we had information on 326 patients with a DAVF-related intracranial haemorrhage. The median age in all study cohorts, including our own, was 54 years (interquartile range (IQR) 51–59 years). The median proportion of men was 73 % (IQR 63–84 %). Eleven studies with a total of 246 patients reported the location of the intracranial haemorrhage: ICH 161 (58 %), SAH 69 (25 %), SDH 16 (6 %), IVH 31 (11 %). In 31 patients the haemorrhage was located in two or more of these compartments.

Treatment modality was not reported specifically for patients with DAVF-related intracranial haemorrhage in most studies. In the total group of 567 patients included in the studies, a total of 556 treatments was performed during follow-up: 388 patients underwent endovascular treatment, 287 patients surgery, 13 patients radiosurgery and 132 patients received multiple treatment modalities. Eleven patients were not treated at all.

Median follow-up for all patients was 12 months (IQR 7–37 months). In the five studies that specified the duration of follow-up specifically for patients with intracranial haemorrhage [15, 23, 26, 28], the median duration of follow-up was 10 months (IQR 6–19 months). The proportion of patients lost to follow-up varied from 0 % to 27 %.

**Fig. 2:**
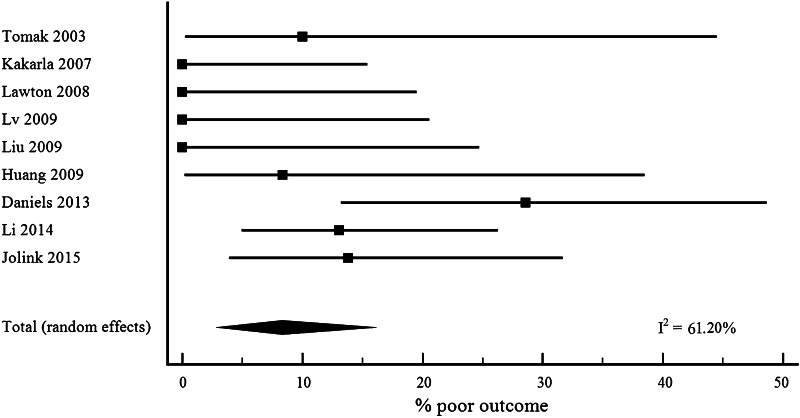
Poor outcome with 95 % CI after DAVF-related intracranial haemorrhage

Case fatality after DAVF-related haemorrhage was 4.7 % (95 % CI 2.5–7.5, 17 cohorts, Fig. 1) [15–30] and the proportion of patients with poor outcome 8.3 % (95 % CI 3.1–15.7; 9 cohorts; Fig. 2) [16, 18, 19, 21–23, 26, 28]. We did not find a statistically significant effect on case fatality for mid-year of the study (β −0.3, 95 % CI −0.8 to 0.2), for mean age (β 0.2, 95 % CI −0.4 to 0.7), for percentage of males (β 0.04, (95 % CI −0.2 to 0.3) or for percentage of patients with an intracerebral component of the intracranial haemorrhage (β -0.1 (95 % CI −0.2 to 0.1). In addition, we found no effect on poor outcome (mid-year of study β 0.4, 95 % CI −1.1 to 2.0; mean age β 0.4, 95 % CI −1.1 to 2.0; percentage of males β 0.4,95 % CI −0.3 to 1.1; percentage of patients with an intracerebral component of the intracranial haemorrhage β 0.1, 95 % CI −0.3 to 0.5).

## Discussion

Hospital based case-series suggest a relatively low risk of death and poor outcome in patients with intracranial haemorrhage due to rupture of a DAVF. We found no association of mid-year of studies, mean age, percentage of males, or percentage of patients with an intracerebral component of the haemorrhage with these outcomes.

In population-based cohorts, patients with an ICH due to an AVM have a risk of death of 12 % after 6 months and of poor outcome at one year of 40 % [8]. This risk is much lower than the reported risk after spontaneous ICH with a risk of death after 6 months of 56 % and of poor outcome at 12 months of 83 % [8]. A possible reason why we found lower estimates of the risk of death and poor outcome for patients with intracranial haemorrhage due to a DAVF is that the studies on which we based our estimates were all hospital based and may therefore have been influenced by referral bias, resulting in underestimation of the proportion of patients with poor outcome. Another explanation may be that in patients with a fatal or severely disabling intracranial haemorrhage due to DAVF rupture, investigations to determine the cause of the intracranial haemorrhage may not always have been performed [31]. Also, DAVFs can be missed on CTA or MRA [32]. A further explanation may be that in contrast to the study that compared outcomes after AVM and spontaneous ICH [8], the cohorts in our study did not restrict inclusion to patients with ICH but included patients with SAH, IVH and SDH as well.

A possibly better clinical outcome from DAVFs in comparison with outcome after spontaneous or AVM-related ICH may be explained by the bleeding site of the ruptured DAVF being venous rather than arterial [26]. Several studies showed that intracranial haemorrhage due to a DAVF is related with cortical venous reflux, venous hypertension and venous congestion [7, 33–35]. Location of the DAVF-related haemorrhage being less often deep may also explain a better clinical outcome [36].

A strength of our study is that by combining the cohorts of two university medical centers and an overview of the current literature, we were able to study a relatively large number of patients given the rarity of this disease and study determinants of outcome. Also, we included patients irrespective of whether they had been treated or not, avoiding the selection mechanisms of some of the previous studies [18–23, 25]. Our study also has limitations. Limitations of our literature search are that none of the included studies fulfilled all our criteria of high quality. Second, outcome in the review includes the effects of treatment and is described over varying durations of follow-up. Furthermore, designs of included studies were heterogeneous, so results need to be interpreted with caution. Third, studies were not always restricted to patients who had presented with intracranial haemorrhage. Ten of the sixteen cohorts described patients treated by a specific modality, most certainly resulting in selection by indication. Also, different ways of reporting functional outcome were used.

Our study has implications for clinical practice. The outcome after the rupture of a DAVF can be used to inform patients and their relatives. Furthermore, our findings should be taken into account when weighing risks and benefits of treatment in patients with unruptured DAVFs. Studies of large patient cohorts, preferably prospective and population based, should give more precise estimates of the risk of poor outcome after DAVF-related intracranial haemorrhage and of factors that may determine outcome.

## Electronic supplementary material

Supplementary material 1 (PDF 119 kb)
