# Supplementary material for: Outcome after intracranial haemorrhage from dural arteriovenous fistulae; a systematic review and case-series
Source: J Neurol. 2015 Sep 26;262(12):2678–83. doi: 10.1007/s00415-015-7898-x (PMC4655013; doi:10.1007/s00415-015-7898-x)
Supplement: Supplementary file 1 — Supplementary material 1 (PDF 119 kb) [file 415_2015_7898_MOESM1_ESM.pdf]

SUPPLEMENTARY MATERIAL

Supplementary figure 1. Flow chart systematic literature search (search date: 16 April 2015)

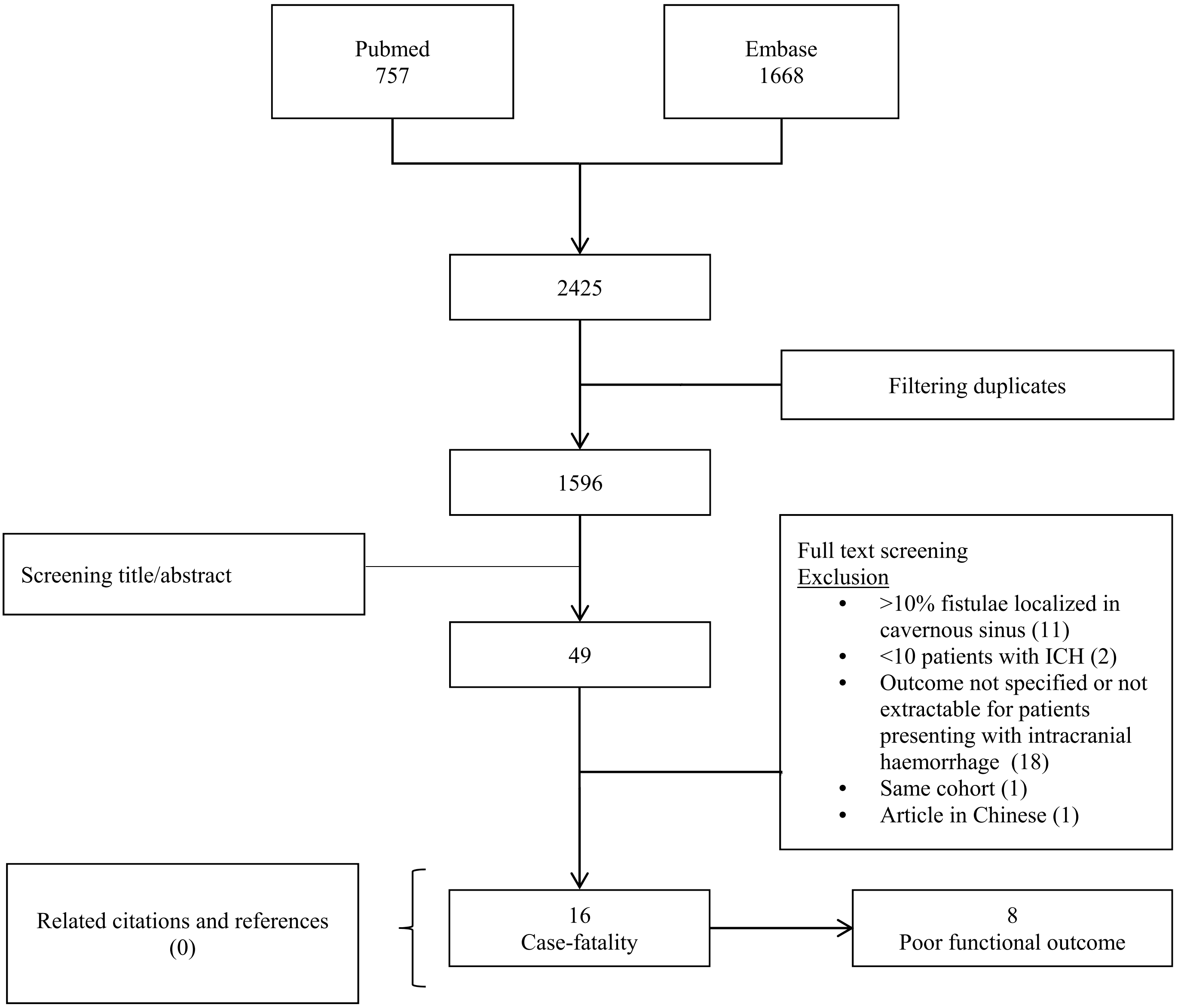

Supplementary table 1. Search strategy for studies of dural arteriovenous fistulae presenting with intracranial haemorrhage

Pubmed:

#1:haemorrhage OR hemorrhage OR hematoma OR haematoma OR bleeding OR haemorrhagic stroke OR hemorrhagic stroke

#2: dura OR dural

#3: arteriovenous fistul* OR arterio venous fistul* OR arteriovenous shunt* OR arterio venous shunt* OR arteriovenous malformation* OR arterio venous malformation* OR arteriovenous fistulous malformation* OR arterio venous fistulous malformation* OR avfistul* OR av shunt* OR avf* OR avfm* OR avs

#4: #2 AND #3

#5: DAVF* OR DAVFM OR ICDAVF OR DAVM*

#6: #4 OR #5

#7: #1 AND #6

Embase:

#1:‘haemorrhage’ OR ‘hemorrhage’ OR ‘hematoma’ OR ‘haematoma’ OR ‘bleeding’ OR ‘haemorrhagic stroke’ OR ‘hemorrhagic stroke’

#2: ‘dura’ OR ‘dural’

#3: ‘arteriovenous’ OR ‘arterio venous’ OR ‘av’

#4: ‘fistula’ OR ‘fistulae’ OR ‘fistulas’ OR ‘shunt’ OR ‘shunts’ OR ‘malformation’ OR ‘malformations’ OR ‘fistulous malformation’ OR ‘fistulous malformations’

#5: #2 AND #3 AND #4

#6: ‘DAVF’ OR ‘DAVFS’ OR ‘DAVFM’ OR ‘DAVFMS’ OR ICDAVF OR ‘ICDAVFS’ OR ‘DAVM’ OR ‘DAVMS’ OR ‘AVF’ OR ‘AVFS’ OR ‘AVFM’ OR ‘AVFMS’ OR ‘AVS’

#7: #5 OR #6

#8: #1 AND #7

Study (First author, year of publication)	Study characteristics			Design	Sample size		Characteristics		Location of intracranial haemorrhage				Treatment					Follow up			Outcome		
	Country of Origin	Inclusion period	Inclusion criteria	Study Design	Total number of patients	Patients with intracranial haemorrhage	Mean age (range) *	Male (%) *	ICH (no. infratentorial)	SAH	SDH	IVH	Untreated	Endovascular	Surgery	Radiosurgery	Multiple	Follow up period (months, range) *	FU period for intracranial haemorrhage patients (months, range)	Patients lost to follow up (%)	Case fatality after intracranial haemorrhage, % (95% CI) †	Predefined outcome	Poor clinical outcome % mRS ≥ 3, GOS ≤ 3 (95% CI) ‡
Jolink 2015	The Netherlands	2007-2012	Presentation with intracranial haemorrhage	RS	29	29	59 (42-74)	17 (59)	18 (11) §	13	0	11	3	22	11	0	7	5 months (0 - 12 months)	5 months (0-12 months)	0	10 (2.2 - 27)	mRS	14 (3.9 - 32)
Al-Mahfoudh 2014	UK, Ireland	2002-2012	Surgical therapy	PS	25	16	57 (23-76)	19 (76)	14 ‖	5	0	1	0	8	25	0	8	67 months (24-102 months)	-	0	0 (0 - 20.6)	-	NRS
Chandra 2014	USA	2006-2012	Endovascular therapy	RS	40	13	57 (30-79)	21 (53)	NR				0	40	0	0	0	28.3 months (12-63 months)	-	27	0 (0 - 24.7)	-	NRS
Li 2014	China	2009-2013	Haemorrhagic DAVFs managed with endovascular therapy	RS	46	46	47 (23-68)	39 (85)	35 #	11	2	8	0	46	0	7	7	20 months	20 months	4	0 (0 - 7.7)	mRS	13 (4.9 - 26.3)
Eftekhar 2013	Australia	1991-2011	Surgical treatment transverse sigmoid sinus DAVFs	RS	42	15	59 (32-78)	20 (48)	NR				0	0	42	0	0	18 months (2-82 months)	-		6,7 (0.2 - 32)	-	NRS
Daniels 2013	USA	1996-2012	Presentation with intracranial haemorrhage	RS	28	28	56,5	24 (86)	20 **	6	5	9	2	20	12	1	7	17 months	17 months	4	10.7 (2.3 - 28.2)	mRS	28.6 (13.2 - 48.7)
Wachter 2011	Germany	1990-2010	Surgical therapy	RS	42	16	58 (35-72)	33 (79)	NR				0	0	42	0	0	50 months (2 - 120 months)	-	0	0 (0 - 20.6)	-	NRS
Guedin 2010	France	1990-2008	ICDAVFs with CVR	RS	53	19	52 (6-85)	35 (66)	13	5	0	1	4	46	4	0	1	3-6 months	-	2	5.3 (0.1 - 26)	-	NRS
Huang 2009	China	2005-2008	TDAVFs with endovascular therapy	RS	14	12	50 (37-65)	13 (93)	0	10 ††	0	4	0	14	0	0	0	3 - 24 months	7.5 months (1-12 months)	0	8.3 (0.2 - 39)	-	8.3 (0.2 - 39)

Liu 2009	USA	1993 - 2007	Surgical therapy	RS	23	13	59 (23-78)	15 (65)	NR				0	12	23	0	12	45 months (2 - 84 months)	-	0	0 (0 - 24.7)	GOS	0 (0 - 24.7)
Lv 2009	China	2005-2008	ICDAVFs with endovascular therapy	RS	40	16	43 (23-60)	29 (73)	NR				0	40	0	0	0	8 months (1 - 16 months)	-	0	0 (0 - 20.6)	mRS	0 (0 - 20.6)
Cognard 2008	France	2003-2006	DAVF' with CVR with onyx therapy	PS	30	16	62 (36-76)	18 (60)	14	2	0	0	0	30	2	2	4	3 months (3-24 months)	-	0	6.3 (0.2 - 30)	-	NRS
Lawton 2008	USA	1997-2006	TDAVFs with microsurgical therapy	RS	31	17	53 (25-87)	21 (68)	11 ‡‡	7	0	0	0	31	31	0	31	50 months (1 - 108 months)	-	0	0 (0 - 19.5)	mRS	0 (0 - 19.5)
Kakarla 2007	USA	1995 - 2004	High risk DAVFs with surgery	RS	53	22	54 (4-85)	37 (70)	NR				0	29	53	1	30	12 months (0 - 84 months)	-	2	0 (0 - 15.4)	GOS	0 (0- 1 5.4)
Van Rooij 2007	The Netherlands	1994-2006	DAVFs Borden type III	RS	29	18	54 (24-77)	24 (83)	16 (4) §§	2	1	0	2	21	12	1	7	6-12 weeks	-	NR	0 (0 - 18.5)	-	NRS
Tomak 2003	USA	1988-2000	Symptomatic TDAVFs	RS	22	10	50 (35-80)	16 (73)	4	5 || ||	0	2	0	20	12	0	10	11.5 months (1 - 123 months)	-	0	10.0 (0.3 - 45)	GOS	10.0 (0.3 - 45)
Duffau 1999	France	1987-1997	ICDAVF's with haemorrhagic presentation	RS	20	20	54 (42-74)	17 (85)	16 (2) ##	3	4	0	0	9	18	1	8	10 months (0.3 - 25 months)	10 months (0.3 - 25 months)	0	15 (3.2 - 38)	-	NRS

Supplementary Table 2. Characteristics of included studies for patients with DAVF presenting with intracranial haemorrhage, including our own cohort of 29 patients

CI indicates confidence interval; CVR, cortical venous reflux; DAVF, dural arteriovenous fistula; GOS, Glasgow Outcome Scale; ICDAVF, intracranial dural arteriovenous fistula; ICH, intracerebral haemorrhage; IVH, intraventricular haemorrhage; mRS, modified Rankin Scale; NR, not reported; NRS, not reported specifically for patients presenting with intracranial haemorrhage; PS, prospective analysis; RS, retrospective analysis; SAH, subarachnoid haemorrhage; SDH, subdural haemorrhage; and TDAVF, tentorial dural arteriovenous fistula.

†Case fatality only for patients with a DAVF presenting with intracranial haemorrhage, ‡Poor outcome (mRS ≥3 or GOS ≤3) only for patients with a DAVF presenting with intracranial haemorrhage, § ICH 7 patients, SAH 6 patients, IVH 3 patients, ICH + SAH 5 patients, ICH + IVH 6 patients, IVH + SAH 2 patients, | | ICH 10 patients, SAH 2 patients, ICH+SAH 3 patients, ICH+IVH 1 patient, # ICH 28 patient, SAH 11 patients, SDH 2 patients, IVH 8 patients, ICH + IVH 7 patients, ** Combination of haemorrhages not reported, †† SAH 8 patients, IVH 2 patients, IVH + SAH 2 patients, ‡‡ ICH 10 patients, SAH 6 patietns, ICH + SAH 1 patient, §§ ICH 15 patients, SAH 2 patients, ICH + SDH 1 patient, | | | | SAH 4 patients, IVH 1 patient, SAH + IVH 1 patient, ## ICH 13 patients, SAH 2 patients, SDH 2 patients, ICH + SAH 1 patient, ICH + SDH 2 patients
